# Renal Cell Carcinoma Discrimination through Attenuated Total Reflection Fourier Transform Infrared Spectroscopy of Dried Human Urine and Machine Learning Techniques

**DOI:** 10.3390/ijms25189830

**Published:** 2024-09-11

**Authors:** Bogdan Adrian Buhas, Lucia Ana-Maria Muntean, Guillaume Ploussard, Bogdan Ovidiu Feciche, Iulia Andras, Valentin Toma, Teodor Andrei Maghiar, Nicolae Crișan, Rareș-Ionuț Știufiuc, Constantin Mihai Lucaciu

**Affiliations:** 1 Department of Urology, Medicover Hospital, 323T Principala St., 407062 Suceagu, Romania; buhasbogdan@yahoo.co.uk (B.A.B.); drnicolaecrisan@gmail.com (N.C.); 2 Faculty of Medicine and Pharmacy, University of Oradea, 1 Universitatii St., 410087 Oradea, Romania; dr.feciche@yahoo.com (B.O.F.); teodormaghiar@yahoo.com (T.A.M.); 3 Department of Medical Education, Iuliu Hatieganu University of Medicine and Pharmacy, 8 Victor Babes St., 400347 Cluj-Napoca, Romania; ana.muntean@umfcluj.ro; 4 Department of Urology, La Croix du Sud Hospital, 52 Chemin de Ribaute St., 31130 Quint-Fonsegrives, France; dr.gploussard@gmail.com; 5 Faculty of Medicine, Iuliu Hatieganu University of Medicine and Pharmacy, 8 Victor Babes St., 400347 Cluj-Napoca, Romania; dr.iuliaandras@gmail.com; 6 Department of Nanobiophysics, MedFuture Research Center for Advanced Medicine, Iuliu Hatieganu University of Medicine and Pharmacy, 4-6 Pasteur St., 400337 Cluj-Napoca, Romania; valentin.toma@umfcluj.ro; 7 Nanotechnology Laboratory, TRANSCEND Research Center, Regional Institute of Oncology, 700483 Iași, Romania; 8 Department of Pharmaceutical Physics–Biophysics, Faculty of Pharmacy, Iuliu Hatieganu University of Medicine and Pharmacy, 6 Pasteur St., 400349 Cluj-Napoca, Romania

**Keywords:** renal cell carcinoma, dried urine, attenuated total reflection Fourier transform infrared, machine learning, linear discrimination analysis–principal component analysis, support vector machine

## Abstract

Renal cell carcinoma (RCC) is the sixth most common cancer in men and is often asymptomatic, leading to incidental detection in advanced disease stages that are associated with aggressive histology and poorer outcomes. Various cancer biomarkers are found in urine samples from patients with RCC. In this study, we propose to investigate the use of Attenuated Total Reflection-Fourier Transform Infrared Spectroscopy (ATR-FTIR) on dried urine samples for distinguishing RCC. We analyzed dried urine samples from 49 patients with RCC, confirmed by histopathology, and 39 healthy donors using ATR-FTIR spectroscopy. The vibrational bands of the dried urine were identified by comparing them with spectra from dried artificial urine, individual urine components, and dried artificial urine spiked with urine components. Urea dominated all spectra, but smaller intensity peaks, corresponding to creatinine, phosphate, and uric acid, were also identified. Statistically significant differences between the FTIR spectra of the two groups were obtained only for creatinine, with lower intensities for RCC cases. The discrimination of RCC was performed through Principal Component Analysis combined with Linear Discriminant Analysis (PCA–LDA) and Support Vector Machine (SVM). Using PCA–LDA, we achieved a higher discrimination accuracy (82%) (using only six Principal Components to avoid overfitting), as compared to SVM (76%). Our results demonstrate the potential of urine ATR-FTIR combined with machine learning techniques for RCC discrimination. However, further studies, especially of other urological diseases, must validate this approach.

## 1. Introduction

Globally, renal cell carcinoma (RCC) ranks as the sixth most commonly diagnosed cancer among men and the tenth among women, constituting 5% and 3% of all cancer diagnoses, respectively [1]. Over the past two decades, the incidence of RCC has generally risen annually by approximately 2% globally and across Europe. In 2022, RCC resulted in 179,368 deaths worldwide, including 115,600 men and 63,768 women, with the global age-standardized rate being 1.8 per 100,000 people [2]. Age and gender are two non-modifiable risk factors, with a gender ratio of 1.5 males to one female and a peak incidence between 60 and 70 years of age [3]. There are two primary adjustable risk factors for kidney cancer: smoking, with a relative risk of 1.58 for those consuming 37.5 packs per year, and obesity, where the relative risk escalates with the body mass index [1,3]. Patients with chronic renal failure are at a significantly higher risk, up to ten times greater than the general population, of developing kidney cancer, often involving both kidneys or multiple sites [4,5].

Many renal masses do not show symptoms until they reach the advanced stages of the disease. The majority of RCCs are discovered incidentally during non-invasive imaging conducted for non-specific symptoms or other unrelated abdominal conditions [6].

Although there is increasing interest in screening programs for RCC, there remains a notable scarcity of research on their effectiveness, cost-efficiency, and the best screening methods. Urinary dipstick tests are not effective as a screening tool due to their low sensitivity and specificity. Additionally, the occurrence of RCC with non-visible hematuria is rare, at just 0.58% [7]. To date, no clinically validated urinary or serum biomarkers for RCC have been established. Computed tomography is not recommended for screening because of its high cost, radiation exposure, and the likelihood of detecting incidental findings. Ultrasound offers acceptable sensitivity and specificity for RCC detection, but its effectiveness can vary depending on the size of the tumor and the skill of the operator. Significant challenges to widespread screening include the low prevalence of RCC, the risk of false positives, and the potential for overdiagnosis of indolent kidney tumors [8,9,10].

Liquid biopsy offers a simpler and less invasive option compared to traditional biopsy methods. Urine, which is straightforward to collect, can yield extensive data useful for detecting cancers and various other diseases. Consequently, the use of urine-based liquid biopsy for diagnosing and monitoring cancer patients has grown in popularity [11].

Recently, the integration of machine learning methods into clinical medicine has significantly increased. This surge is mainly attributed to the enhanced power and a broader understanding of the benefits these methods bring to clinical environments. Such approaches have notably improved disease diagnosis and enabled the development of advanced care strategies for patients [12].

Vibrational spectroscopy provides insights into the molecular structure, composition, and dynamics of samples without requiring tags or dyes [13,14]. There has been a notable increase in the use of Fourier transform infrared (FTIR) spectroscopy for analyzing biofluids such as saliva, blood, and urine, as well as tissues and cells [13,15,16,17,18,19,20,21]. However, most studies published in this field were focused on the FTIR analysis of dried blood components [22] and a significantly lower number of studies were dedicated to the FTIR analysis of dried urine.

In fact, among others (accuracy, costs, time consumption, the need for specialized personnel, etc.), a major problem in putting a screening method into practice for a disease is its acceptance by the patients. In this sense, urine tests are more accepted by patients as compared to blood tests. Collecting a urine sample is usually less time-consuming and more convenient compared to blood tests, as it does not require venipuncture or a visit to a medical professional. Patients can often collect urine samples in the privacy of their own homes. Since urine tests are noninvasive and do not involve needles or puncturing the skin, they are perceived as less painful and uncomfortable by many patients, leading to higher acceptance rates. For these reasons, we tried, in this paper, to evaluate the capacity of Attenuated Total Reflection Fourier Transform Infrared (ATR-FTIR) spectroscopy of dried human urine combined with machine learning techniques to discriminate RCC. Previous studies have successfully quantified urinary urea, protein, creatinine, and cysteine using urine infrared spectra [21]. Research utilizing urine has also been directed toward investigating cancers including endometrial, ovarian [23], prostate [24], and esophageal adenocarcinoma [25]. ATR-FTIR spectroscopy has proven useful in forensic science [26] and in identifying specific spectral markers of renal injury in rodent models with acute and progressive glomerulonephritis [17]. Additionally, FTIR has been used to analyze urea in animal urine studies [27]. In many cases, chemometric methods have been employed to distinguish subtle differences between diseased and control groups due to overlapping spectral bands. 

In a previous study [28], some of us investigated the utility of label-free Surface-Enhanced Raman Scattering in urine, coupled with two machine learning approaches: Principal Component Analysis combined with Linear Discriminant Analysis (PCA–LDA) and Support Vector Machine (SVM), to discriminate between RCC patients and healthy donors. Employing LDA-PCA, we achieved a discrimination accuracy of 100% using 13 principal components. The SVM approach yielded a training accuracy of 100% and a validation accuracy of 99% for discriminating between RCC and control. We also showed that the selection of Principal Components with markedly distinct scores between the two classes serves to alleviate overfitting risks and reduces the number of components employed for discrimination. 

In this paper, we present the results obtained in the discrimination of the RCC cases from the controls by using ATR-FTIR of dried urine samples using both LDA-PCA and SVM. We also performed an analysis of the ATR-FTIR features of urine components aiming at increasing the assignment accuracy of the major ATR-FTIR bands of human urine and understanding which molecules contribute mostly to the discrimination between the healthy donors and patients’ urine samples. We compare the PCA–LDA and SVM approaches in terms of the accuracy of discrimination. This approach allows us also to compare the accuracy achieved in the discrimination figures of RCC cases using ATR-FTIR and SERS, two complementary vibrational spectroscopy techniques. A key difference between the two vibrational techniques lies in how signal intensities are measured. In FTIR, the absorption is directly related to the concentration of molecular species present in the sample. However, in SERS, the intensity of scattered signals depends on the Raman cross-section of the molecule and its affinity to the SERS substrate. As a result, SERS spectra may sometimes be dominated by species with lower concentrations. We will explore how these differences impact the discrimination ability of the two techniques.

## 2. Results

### 2.1. ATR-FTIR Spectra of Urine Samples: Comparison with Artificial Urine and Urine Components

Urine is a complex fluid primarily composed of urea (10–35 g/day per capita) and smaller amounts of creatinine (1–1.8 g/day per capita), uric acid (0.25–0.80 g/day per capita), and creatine (0–0.15 g/day per capita) [29]. It also contains phosphate, sodium, potassium, and ammonia. Typically, urine has low protein levels, often considered protein-free or containing only trace amounts (<0.15 g/day per capita) [30]. Higher protein levels, known as proteinuria, indicate kidney disease or other conditions and are usually measured by the protein-to-creatinine ratio [31].

**Figure 1:**
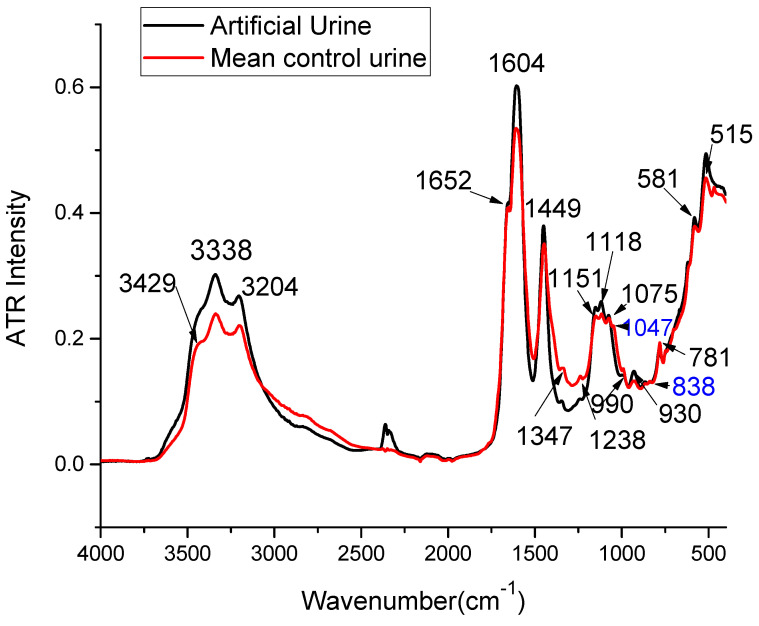
ATR-FTIR spectrum of artificial urine (black) and the mean spectrum obtained from the urine of 39 control patients (red). The wavenumbers corresponding to the main peaks in the two sets of spectra are also indicated in cm^−1^.

We present, in Figure 1, the mean ATR-FTIR spectrum of urine samples collected from healthy donors in comparison with the spectrum obtained from artificial urine prepared, as described in Section 2.3. As can be observed from this figure, almost all features observed in the ATR-FTIR in human urine are also noticed in the spectrum of artificial urine. In the high wavenumber region, two main peaks were recorded at 3204 cm^−1^ and 3338 cm^−1^, and a shoulder at 3428 cm^−1^. Other major peaks were recorded in both spectra at 1604 cm^−1^ and 1449 cm^−1^, in the fingerprint region, and a shoulder at 1652 cm^−1^. Other clearly distinguishable peaks were recorded at 1151 cm^−1^, 1118 cm^−1^, 1075 cm^−1^, 990 cm^−1^, 930 cm^−1^, and 781 cm^−1^. Below 750 cm^−1^, the background signal increases significantly, which makes it more difficult to investigate the peaks. However, two peaks at 581 cm^−1^ and 515 cm^−1^ are clearly identifiable. Two smaller peaks recorded at 1047 cm^−1^ and 838 cm^−1^ were not observed in the artificial urine spectrum.

**Figure 2:**
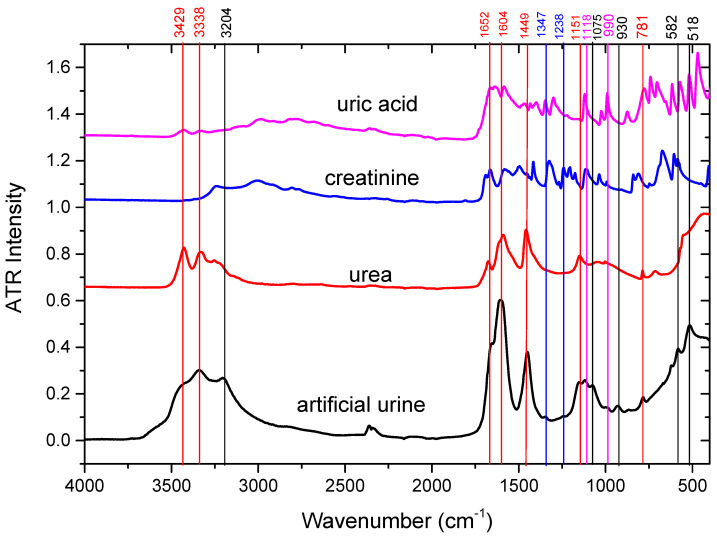
Comparison of the ATR-FTIR spectrum of artificial urine (black) with the spectra of the main organic urine components: urea (red), creatinine (blue), and uric acid (magenta). The vertical lines were traced to help identify the peaks of artificial urine with the peaks of the three components. The line and peak wavenumber colors indicate the compound for which we have the best match, and the black lines are traced for artificial urine peaks not matching the peaks of urea, creatinine, or uric acid.

Aiming at assigning the various peaks observed in urine, in Figure 2 we represented the ATR-FTIR spectra of artificial urine and its main organic components: urea, creatinine, and uric acid. As can be noticed from this figure, the urine spectrum resembles mostly that of urea, which is its major component, with major peaks at 3338 cm^−1^, 1604 cm^−1^, and 1449 cm^−1^. The shoulder at 1652 cm^−1^ matches with a peak of creatinine but is close to maximum absorption peaks of uric acid and urea. This kind of superposition is also observed for other peaks so that one cannot unequivocally assign these peaks to only one compound. For this reason, in Table 1, we provide the wavenumbers corresponding to the major peaks measured in urine ATR-FTIR spectra together with the assignments proposed in the literature and from the present study.

In some cases, the absorption peaks of urine do not match with peaks from these three major urine compounds, and the corresponding vertical lines are traced in black. In this case, the possible assignments were made based on the ATR-FTIR spectra of other urine compounds: citrate (1575 cm^−1^), sulfate, and phosphate (1075 cm^−1^, 930 cm^−1^) (Figure S2).

The two peaks recorded at around 2350 cm^−1^ correspond to the asymmetric stretching vibrations of the atmospheric CO_2_ molecule.

**Figure 3:**
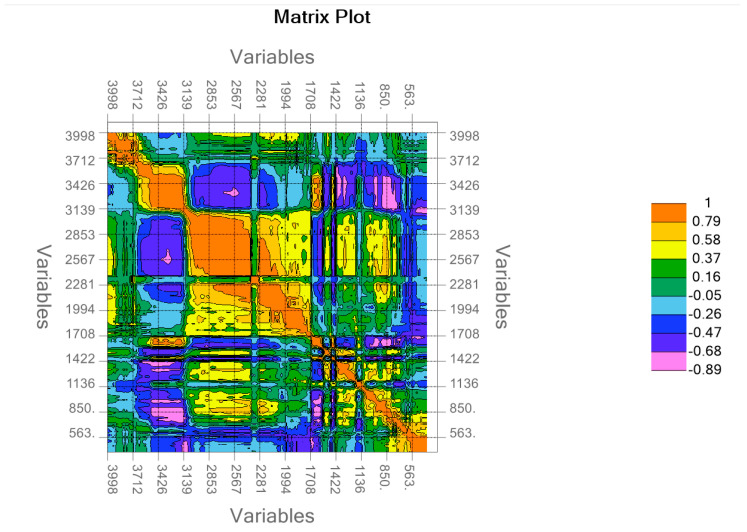
Matrix plot of the correlations between the ATR-FTIR absorption intensities measured for all the urine samples.

As can be seen in this table, for some peaks, simply comparing the IR absorption of urine with that of major urine components cannot provide unambiguously its source, due to the superpositions. To obtain more insights into the assignments, we traced the matrix correlation plot of the ATR-FTIR absorption intensities (Figure 3).

It has to be mentioned that this matrix correlation plot is related to the intensities measured at different wavenumbers without considering if these wavenumbers correspond or not to peaks in their respective spectrum. From this plot, we noticed that the ATR-FTIR intensities correlate in some close ranges (e.g., 3200–3400 cm^−1^, 2400–2900 cm^−1^, 1600–1700 cm^−1^). Long-range correlation can be observed between the wavenumber range 1600–1700 cm^−1^ and three areas in the wavenumber range 3150–3450 cm^−1^. These correlations might be assigned to urea which presents absorption maxima in these two spectral ranges. Due to the limitations of this approach, we also represented all the urine spectra in a multiple scatter form using the Unscrambler software. Focusing on the most intense ATR-FTIR peaks (not for all wavenumbers as plotted in Figure 3), we present, in Figures S3 and S4, the regression curves and the corresponding coefficients of determination (R^2^) for the most intense vibrational bands of urine. The 1604 cm^−1^ assigned to urea presents the highest correlation (R^2^ ~ 0.92) with the peak at 1447 cm^−1^ and an R^2^ = 0.89 with the shoulder measured at 1652 cm^−1^. Also, we noticed a high coefficient of determination (R^2^~0.98) between the peaks recorded at high wavenumbers 3338 cm^−1^ and 3429 cm^−1^. Lower values of the coefficient of determination (range 0.55–0.7) between the peaks recorded at high wavenumbers and those in the fingerprint region (Figure S3). In the case of the peaks assigned to creatinine (Figure S4), there is a coefficient of determination of 0.73 for the linear regression between the ATR-FTIR intensities measured at 1118 cm^−1^ and 1238 cm^−1^ assigned to creatinine. However, lower values of 0.46 and 0.52 were obtained when the intensity of the peaks, as mentioned above, was compared to the intensity of the shoulder recorded at 1652 cm^−1^. The latter shoulder intensity seems to correlate better with the peaks assigned to urea. Also, the peak measured at 3204 cm^−1^ has a high coefficient R^2^ = 0.82 with the peak recorded at 3429 cm^−1^, assigned to urea, and an R^2^ = 0.76 with the shoulder measured at 1652 cm^−1^. Nevertheless, it has to be mentioned that, because urea is much more concentrated in urine as compared to creatinine, the higher the concentration of a compound in a mixture the more important its contribution to the IR absorption of the mixture, although, in some cases, this absorption occurs at frequencies which are specific for lower concentrated compounds.

As can be seen in Figure 2, many features in the IR spectra of creatinine and uric acid are masked by the more important absorption of urea, which is the most concentrated compound in urine. The observation is valid for citrate and phosphate groups (Figure S2).

To obtain more insights into the assignment of the vibrational bands we used another approach, namely, measuring the ATR-FTIR of artificial urine spiked with urea, creatinine, uric acid, and citrate and following the changes in the absorbances at different wavenumbers (Figure S5). Once again, all the spectra are dominated by urea absorption, which remains the most concentrated compound. However, we can also notice some slight changes for each spiked urine sample. In the case of citrate, the maximum recorded in urine at 1604 cm^−1^ is shifted to 1586 cm^−1^, probably due to the strong absorption peak of this compound at 1575 cm^−1^. However, no distinct peak at the latter wavenumber can be distinguished. A distinct peak at 1391 cm^−1^ is evidenced in the citrate-spiked artificial urine. For creatinine-spiked artificial urine, the two peaks at 1241 cm^−1^ and 1340 cm^−1^ situated very close to the peaks detected in urine at slightly different frequencies (1238 cm^−1^ and 1347 cm^−1^, respectively), significantly increas their intensities; based on this observation, we assigned them to creatinine. A distinct peak at 1487 cm^−1^ is evidenced in the creatinine-spiked artificial urine which cannot be observed in the artificial or human urine, probably due to it being masked by the strong absorption of urea. In the case of uric acid spiked artificial urine, a peak at 1118 cm^−1^ matches a small peak recorded at the same wavenumber in artificial urine and might be assigned to this compound.

In the very-low wavenumber range, the two peaks at 518 cm^−1^ and 582 cm^−1^ cannot be observed in the urea-spiked artificial urine, meaning that they cannot be assigned to urea but are masked by the strong urea absorption. However, no definite assignment can be provided for these peaks as they appear in all the other spiked-urine samples. Based on the measured compounds and the literature data, we tentatively assign them to glucose and phosphate, respectively.

### 2.2. ATR-FTIR Spectra of RCC Patients’ Urine Versus Healthy Donors

**Figure 4:**
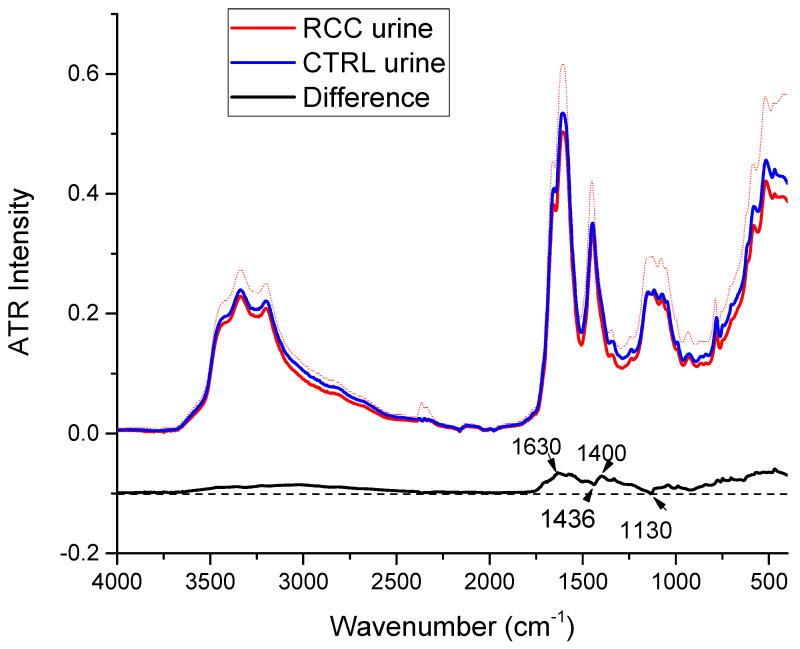
The mean ATR-FTIR spectrum of urine from the RCC patients (red) and the healthy donors (CTRL) (blue) and the difference between the two mean spectra (black). Dashed areas represent the standard deviations. The difference spectrum was offset for better visualization.

The mean ATR-FTIR spectra for the 44 RCC patient samples and 39 healthy donor ones (CTRL), together with their standard deviations and the difference spectrum, are presented in Figure 4. The ATR-FTIR spectra of all samples from both control subjects and RCC patients are provided in Figure S5. While looking at all spectra in Figure S5 one cannot notice a clear-cut difference between the two groups; a slight difference might be observed in Figure 4, where, for almost all wavenumbers, the ATR-FTIR intensity seems to be higher for the CTRL group. The difference spectrum present some positive and negative peaks at 1632 cm^−1^, 1400 cm^−1^, 1036 cm^−1^, and 1130 cm^−1^ (see Figure 3), which do not match major peaks in the urine or urine components spectra.

As shown in Figure 4, where the standard deviations are represented as shaded areas, there is no significant difference between the ATR-FTIR intensities of the two groups as the standard deviations overlap. Moreover, in the lower wavenumber range (below 750 cm^−1^) the standard deviations increase significantly for both groups, indicating a large variability between all the samples.

However, we performed the Student’s *t*-test on all peaks identified in the ATR-FTIR spectra of urine. The test results, performed with the Unscrambler program for a 95% confidence level (described as α = 0.05), are summarized in Table 2, where *p* values are provided for each peak.

The results show a statistically significant difference and a highly significant difference for the peaks at 1347 cm^−1^ and 1238 cm^−1^, respectively, which are both assigned to creatinine. For both peaks, the ATR-FTIR intensities are higher in the CTRL samples as compared to the RCC ones. Also, a significant difference between the two groups was obtained for the shoulder recorded at 1652 cm^−1^, which was assigned to both urea and creatinine. Lower creatinine in urine could be associated with impaired kidney function, which is quite common in RCC.

Another notable observation is that individual spectra reveal several additional peaks not listed in Table 1. For instance, in the spectral range of 2800–2900 cm^−1^, which is characteristic of CH and CH₂ symmetric and/or asymmetric stretching, no distinct peaks are observed in the average spectra (Figure S6). However, these peaks are clearly visible in individual samples, as we present in Figure S7, in which we represented the absorbances in the spectral range of 2800–2900 cm^−1^

The infrared spectrum of a bodily fluid, like urine, offers extensive information by detecting all active molecular groups simultaneously. However, a significant challenge arises from closely spaced absorption bands that often overlap, creating large envelopes and making it difficult to discern small spectral changes. To extract meaningful information from this complex spectral data, the simple statistic tests have a limited application, and therefore data mining techniques are essential.

### 2.3. PCA–LDA Analysis

The FTIR spectra of urine samples show that the spectral features in the mid-infrared region are concentrated in specific wavenumber ranges. Consequently, we excluded from our analysis the spectral regions without IR absorption for urine and its components. In the high wavenumber range, we analyzed the interval from 3500 cm^−1^ to 2500 cm^−1^. In the low wavenumber range, we focused on from 1750 cm^−1^ to 700 cm^−1^. Although some characteristic peaks were observed below 700 cm^−1^, the data showed significant variability, so we chose not to include this range in our analysis. In this manner, our data set consisted of 88 samples and 1435 variables.

Before applying the multivariate analysis, we proceeded with preprocessing the spectra. In the first step, the spectra were smoothed using the Savitzky Golay algorithm, and, afterward, we applied a detrending algorithm. The individual spectra of all samples provided in Figure S8, show that these transformations decrease the variability of the data, which might be produced by the spectrometer. Also, in Figure S9, we present the mean and standard deviation for the two groups. From the visual inspection of this figure, it seems that the preprocessing steps decrease the differences between the two groups.

Considering the large number of variables recorded, we performed, in the first step, an unsupervised Principal Component Analysis (PCA) which aimed at reducing the dimensionality. We reduced the dimensionality to 15 principal components (PCs), capturing over 99% of the data variance. The explained variance for these PCs is detailed in Figure S10, with loading plots shown in Figure S11. The model was validated using the leave-one-out cross-validation (LOOCV) method, which systematically uses one sample as a validation set while training on the rest, ensuring comprehensive model evaluation. Notably, the first PC explains more than 64% of the variability, the second 14%, and the third and fourth PCs account for 7% and 4%, respectively. To identify principal components (PCs) with significantly different scores between the two groups, we conducted a Student’s *t*-test on all 15 PCs. The test results, performed with the Unscrambler program for a 95% confidence level (described as α = 0.05) are summarized in Table 3, where the *p* coefficient is provided for each PC.

**Figure 5:**
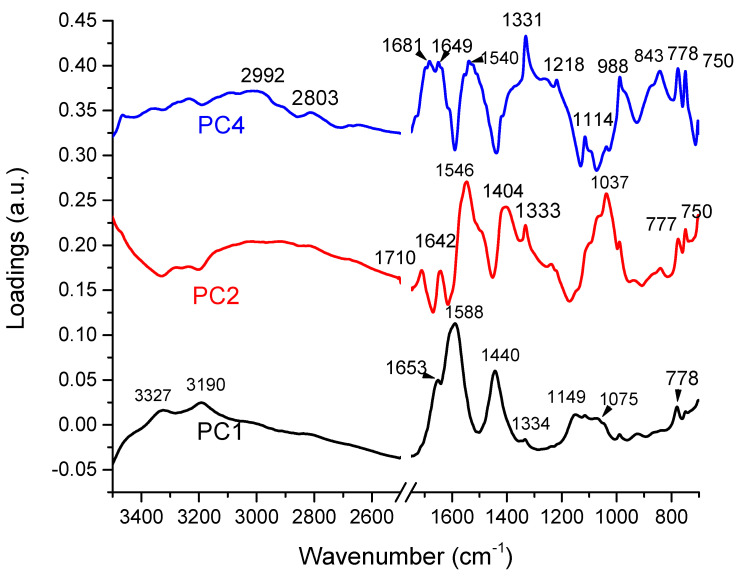
Loading plot for PC1 (black), PC2 (red), and PC4 (blue).

In Figure 5 we present a much more detailed image of the loadings corresponding to the first two PCs, which explain 78% of the variance, and for PC4, which has highly significantly different scores (*p* < 0.001) between the two groups in the Student’s *t*-Test. The loading curve for PC1 closely resembles the FTIR spectrum of urine, with slight differences in wavenumbers, and explains most of the variance due to urea’s dominance. For PC2, negative peaks appear in the high wavenumber range (3000–3500 cm^−1^), and there are three major peaks at 1546 cm^−1^, 1404 cm^−1^, and 1037 cm^−1^, which do not match major urine components. Peaks at 1642 cm^−1^ and 1710 cm^−1^, associated with carbonyl, CC double bond, and amide I vibrations, are likely masked by urea but revealed through this analysis.

PC4’s loading curve, showing significant differences between RCC and CTRL samples, features CH stretching vibrations (2800–2900 cm^−1^) in the high wavenumber region, potentially linked to lipids and other molecules. In the fingerprint region, three peaks recorded at 1331 cm^−1^, 1218 cm^−1^, and 1114 cm^−1^ might be associated with creatinine. As presented earlier, the peaks assigned to creatinine have a significantly lower ATR-FTIR intensity as compared to control samples. Other major peaks measured at 1681 cm^−1^, 1649 cm^−1^, and 1540 cm^−1^, might be associated with proteins, but also with carbonyl groups, like in ketone bodies. However, the FTIR method is limited and cannot provide clear-cut information about the exact chemical nature of the molecules absorbing at a specific wavenumber.

In Figure S12, we present a 2D plot of the scores using PC4 and PC2 where we can notice a slight separation between the two groups. It is obvious that two PCs alone do not completely discriminate between the groups, indicating the need for more components, especially in an unsupervised analysis. Subsequently, we systematically applied a supervised classification method to distinguish between the datasets.

We employed Discriminant Analysis combined with Principal Component Analysis (LDA-PCA). The analysis considered up to 15 principal components (PCs) and utilized three discrimination functions: linear, quadratic, and Mahalanobis. To evaluate the model’s performance, we used the same leave-one-out cross-validation (LOOCV) method as before, but now in a supervised context. Each sample was compared with others, considering the category of the samples.

**Figure 6:**
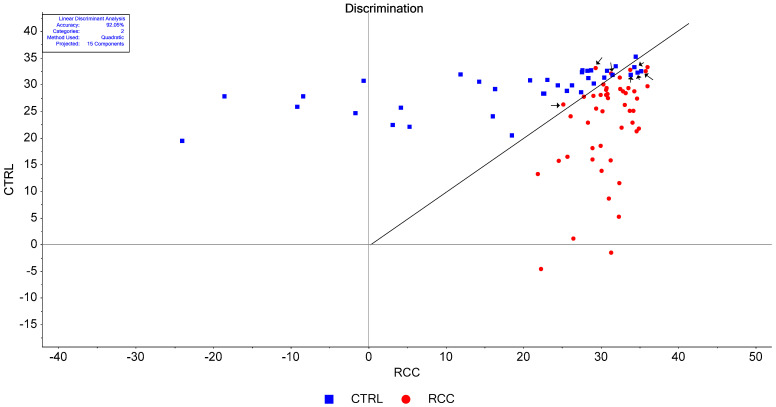
Discrimination plot between the RCC and CTRL samples using a quadratic discrimination function and taking 15 PCs. For each sample, the software provides a score for the two groups CTRL and RCC. The sample is assigned to the group for which the score is highest. From a graphical point of view, the bi-dimensional space is split in two by the bisector of the first quadrant. The data points situated to the right from this bisector belong to the RCC group and the data points situated to the left from this bisector are assigned to the CTRL group. One can notice that three RCC cases (red circles) were assigned to the CTRL group (False Negative) and four CTRL samples (blue squares) were assigned to the RCC group (False Positive). The misassigned samples were marked with arrows. From 88 samples, 81 were assigned correctly, i.e., the accuracy was 92.05%.

**Figure 7:**
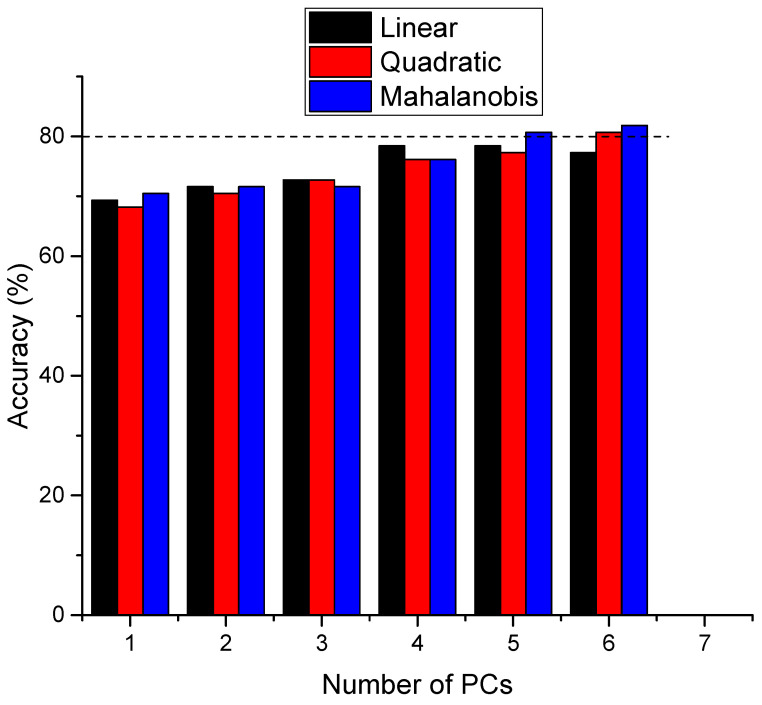
Accuracy of discrimination between the RCC and CTRL samples as a function of the number of PCs considered for the linear, quadratic, and Mahalanobis functions. The PCs were chosen in the order of their difference between the two groups (increasing the *p*, Pearson’s coefficient, from the Student’s *t*-Test, Table 3).

A discrimination plot using all 15 PCs and a quadratic discrimination function is presented in Figure 6, and the corresponding confusion matrix is presented in Table S4. The main issue with this type of analysis is the number of PCs that are taken into consideration when performing the calculations. As the number of PCs increases the accuracy figures increase in their turn, and there is no clear-cut criterion to establish a correct number of PCs. Some heuristic methods were proposed for correctly choosing the number of PCs [41]. For example, according to the elbow rule, the number of PCs to be considered in PCA corresponds to the PC at which the explained variance curve (Figure S8) levels off, meaning that adding more PCs will not add much-explained variance. Another possibility is to observe the PC number corresponding to the sudden change in the slope of the eigenvalues represented against the number of PCs in the so-called Scree Plot (Figure S13). However, this method was criticized as, in many cases, the two curves do not resemble an elbow, and therefore it is difficult to decide the number of PCs. In our case, as can be seen in both figures (Figures S10 and S13), the tradeoff between the number of PCs and the accuracy values is somewhere between PC3 and PC6. For this reason, we systematically investigated the accuracy figures with a variable number of PCs, up to six PCs, using all three discrimination functions (Figure 7). Moreover, based on our previous experience [28] in LDA-PCA, we considered the PCs in the order of their *p* values in the Student’s test (Table 3).

As expected, the discrimination accuracy increases with the number of components considered in the analysis, reaching values above 80% for the nonlinear discrimination functions and six PCs. However, we believe that this accuracy level is high enough for clinical applications.

### 2.4. Support Vector Machine

Support Vector Machine (SVM) classification, like Linear Discriminant Analysis (LDA), is widely used in data mining for pattern recognition. We applied the SVM algorithm to our data using The Unscrambler^®^ software, which utilizes code developed by Chang and Lin [42]. SVM seeks to find an optimal hyperplane to separate different classes. When a linear separation is not possible, SVM employs a kernel function to map data into a higher-dimensional feature space, allowing linear algorithms to operate efficiently in this transformed space, thus handling non-linear data effectively. The method is implemented in the Unscrambler X 10.5.1 software through a linear, a polynomial, a sigmoid, and a Radial Basis Function (RBF).

For optimizing the parameter, in the sense of increasing the discrimination accuracy, a coarse grid search was initially conducted, followed by a more detailed fine grid search. The cross-validation method used was an 88-segment cross-validation, equivalent to leave-one-out cross-validation (LOOCV). In this approach, each sample was sequentially tested using a classifier trained on the remaining 87 samples. This method helps prevent overfitting by ensuring that each sample is tested on a model trained without it, allowing for an unbiased assessment of the classifier’s performanc, The discrimination accuracies obtained by using four kernel functions are presented in Table 4.

For all kernel types, the training accuracy is noticeably higher than the validation accuracy. Interestingly, for both the linear and polynomial kernels, the training accuracy is 88.6%, while the validation accuracy remains consistent at 76.1%. In the case of RBF, there is the largest difference between the training and validation accuracies, which is an indication of overfitting. In the case of the sigmoid kernel, we calculated the worst accuracy values for both training and validation, which indicates that this kernel is not suitable for this type of data set.

Comparing the two machine learning methods, it seems that PCA–LDA surpasses Support Vector Machine (SVM) in generating discrimination scores, particularly in highlighting subtle yet significant differences. Beyond offering superior discrimination scores, PCA–LDA identifies the most influential components, providing valuable insights into the metabolites responsible for the observed distinctions. This detailed information is essential for understanding the biochemical basis of group differences, enabling more thorough and meaningful interpretations.

## 3. Discussion

While imaging techniques such as ultrasound, computed tomography (CT), and magnetic resonance imaging (MRI) remain the cornerstone for detecting renal masses, these methods have several limitations when applied to routine screening. CT, despite its high sensitivity, involves significant radiation exposure, making it unsuitable for repeated use, especially in asymptomatic individuals. Ultrasound is more widely available and avoids radiation, but its diagnostic accuracy can vary depending on the operator and the size of the tumor. MRI, while offering excellent soft-tissue contrast, is expensive and not feasible for large-scale screening programs [1,2,43].

Liquid biopsy and urine-based biomarkers have also gained interest in RCC detection due to their non-invasive nature. However, while promising biomarkers like carbonic anhydrase IX (CA9), aquaporin-1, and microRNA panels have shown potential, they still face challenges regarding sensitivity, specificity, and clinical validation. Biomarker concentrations can fluctuate depending on disease progression, patient characteristics, or even sample handling, making it difficult to establish standardized thresholds for clinical use. Furthermore, no single biomarker has demonstrated sufficient sensitivity or specificity to be recommended as a reliable tool for RCC screening or diagnostic [3,44,45,46,47].

FTIR analysis of urine is a powerful diagnostic and monitoring tool for various diseases. It is widely used in nephrology for detecting conditions like nephritis [17], pathogenic bacteria in urine [20], cystinuria [21], and chronic kidney disease [32]. Studies also suggest using urine FTIR for monitoring diabetes by detecting increased glucose levels in urine [34] and screening for autism spectrum disorder [48]. Given the prevalence of cancer, urine FTIR has been proposed for diagnosing various cancers, including ovarian [24], urinary bladder [39], and prostate cancers [49].

Some of us previously utilized Surface-Enhanced Raman Scattering (SERS) on serum [50] and urine [28] to differentiate RCC from healthy donors. The performance metrics for urine SERS were slightly lower than those for serum SERS. In serum, we achieved 100% discrimination accuracy using PCA–LDA with 12 PCs and a quadratic discrimination function, while, in the case of urine, the same accuracy was obtained for 15 PCs. This difference may be due to serum’s higher content of molecules like uric acid and hypoxanthine, with high affinity to the SERS substrates, which vary in concentration between the groups. Urine SERS discrimination mainly relied on differences in urea and creatinine levels [28] but the urine SERS peaks were also assignable to hypoxanthine and uric acid. In fact, the intensity of SERS signals relies not only on the Raman cross-section of the molecules of interest but mostly on their affinity for the metallic substrate. In their pioneering works, Bonifacio et al. [51] systematically investigated the SERS of blood components (serum and plasma) on different substrates for diagnostic purposes and they demonstrated that the most intense peaks belong to purinic metabolites, which might have diagnostic values, especially in cancers. We have to mention that, for some SERS substrates, the spectra are dominated by proteins, hindering the SERS effect for lower molecular mass compounds and involving the need for a deproteinization step. However, it was also demonstrated that, with a proper choice of SERS substrate, the deproteinization step is not needed [52,53], making SERS a very powerful technique in disease diagnosis.

In this study, we investigated the use of urine ATR-FTIR for distinguishing RCC. Initially, we aimed to accurately assign the major vibration peaks of urine samples by comparing their spectra with those of major urine components and artificial urine. We found a strong correlation between the spectra of artificial urine and actual urine samples. Our findings, including tests with artificial urine spiked with various components, showed that urine FTIR spectra are primarily dominated by urea, with smaller intensity peaks attributable to creatinine and minor contributions from components like uric acid and phosphate. This important contribution of urea to the ATR-FTIR signal of urine might mask the effects of other low-molecular-mass compounds which might be relevant for discrimination or cancer cases.

By comparing the average urine spectra of the RCC and CTRL samples and conducting statistical analysis, we identified only two small peaks with statistically significant differences, both associated with creatinine. The dominance of urea in urine FTIR spectra, which masks peaks in other constituents, likely explains the lower discrimination performance between cancer and non-cancer samples using FTIR compared to SERS. We believe that for future studies one could reduce the contribution of urea by reducing the weights of the variables corresponding to the wavenumbers with high urea absorbances in the multivariate analysis.

In the PCA–LDA analysis, we demonstrated that, by selecting the principal components (PCs) with the most significant differences between cancer and healthy donor samples, we can reduce the number of PCs needed while increasing discrimination accuracy. This approach allows for a more efficient and accurate differentiation between the two groups. Our study also demonstrates that Principal Component Analysis–Linear Discriminant Analysis (PCA–LDA) outperforms Support Vector Machine (SVM) in distinguishing between cancer and healthy donor samples. PCA–LDA not only provides superior discrimination scores but also might identify some key metabolites responsible for the observed differences, thus aiding in understanding the biochemical basis of these distinctions. While SVM can identify support vectors, it is less intuitive to link them to FTIR spectra compared to PCA–LDA.

Although the discrimination figures obtained in this study are smaller as compared to those obtained by SERS, we still believe that urine FTIR has huge potential in the clinical laboratory. FTIR spectroscopy is notable for its ease of use, minimal laboratory space requirements, and lower setup and operating costs. It requires only a small droplet of biofluid (microliters) for analysis, and samples can be analyzed directly without needing tags or labels, and the recorded spectra do not rely on a specific substrate like in the case of SERS. One limitation of our study is the limited number of samples included. More data including other types of cancers are needed before considering translation into the clinic. Also, improving the machine learning techniques could eventually improve the discrimination figures. Despite these challenges, our findings indicate that urine analysis shows significant potential for disease diagnosis, particularly in kidney cancer.

## 4. Materials and Methods

### 4.1. Research Ethics

All participants provided informed written consent according to the Declaration of Helsinki 2013. The study protocol was approved by Cluj-Napoca Municipal Clinical Hospital, Ethics Committee Decision No. 1 from 19 January 2018, for the study entitled “Biomarkers for early diagnosis of bladder, prostate, and kidney cancer by Raman spectrophotometric profile analysis of biological fluids (blood and urine) and humoral tissues involving humans”.

### 4.2. Cohort of Patient Samples and Urine Collection

A total of 118 samples were collected from individuals (both males and females) diagnosed with renal tumors, as confirmed by contrast-enhanced computed tomography (CECT), with informed written consent obtained from each participant. Samples from RCC patients were taken before any surgery or treatment. To minimize sample variability, this study focused on data from 49 male patients with RCC, confirmed through post-surgery histopathological examination. This study also included 39 samples from apparently healthy volunteers, who had provided informed written consent and visited the hospital for routine check-ups without any history of kidney diseases, attempting to match the age distribution of the patient samples. The age range for RCC patients was from 38 to 78 years, while, for healthy donors, it was from 19 to 88 years. Demographic and statistical details are provided in Tables S1–S3.

### 4.3. Reagents and Instrumentation

Artificial urine was prepared according to reference [54] and with the composition indicated in Table S4. The reagents used for obtaining artificial urine were as follows: urea, uric acid, and tri-sodium dihydrate from VWR, (Leuven, Belgium), anhydrous creatinine >98%, monobasic and dibasic sodium phosphate from Sigma-Aldrich (St. Louis, MO, USA), and the other salts were of analytical grade from a local provider (Chemical, Iasi, Romania).

FTIR spectra were recorded with a Bruker TENSOR II instrument (Bruker Optics Inc., Billerica, MA, USA) in attenuated total reflectance mode using the platinum attenuated total reflectance (ATR) accessory in the 400–4000 cm^−1^ spectral range with a resolution of 4 cm^−1^.

### 4.4. Urine Deposition and ATR-FTIR Measurements

In the initial step, we tested the volume of urine to obtain reproducible and high-intensity ATR-FTIR spectra. Our results showed that 2 μL is the optimum volume; for lower volumes the FTIR signal decreases in intensity, and for larger volumes no significant increase in the intensity was noticed, instead, a longer period was needed to wait until the samples dried. In ATR, the evanescent wave penetrates the sample beyond the internal reflection element (in this case, diamond), with a typical penetration depth of from 1 to 2 μm in the 1800–900 cm⁻^1^ range. Increasing the volume of the sample deposited on the ATR crystal may increase the thickness after drying, and consequently the intensity of the ATR signal. Beyond a certain point, the ATR signal no longer increases due to the limited penetration depth of the evanescent wave [55].

For each sample, 2 μL of urine was deposited on the diamond crystal of the spectrometer and allowed to dry, and two consecutive spectra of 16 scans each were recorded. The ATR diamond was then thoroughly cleaned with isopropyl alcohol, allowed to dry, and another 2 μL of urine was deposited. This process was repeated three times for each sample, with the FTIR spectra being recorded twice for each deposition. This means that for each sample a minimum number of six spectra were recorded and averaged for further use in the statistical analysis. The background was recorded on the clean diamond crystal every 10–15 min. An example of such ATR-FTIR spectroscopy recordings is presented in Figure S1. As can be seen from Figure S1, the spectra recorded for a given deposition are almost overlapping. However, there are slight differences between the ATR-FTIR intensities measured between different depositions mostly in the very low wavenumber range.

### 4.5. Multivariate Analysis

Multivariate analysis of ATR-FTIR spectra was performed using Unscrambler X 10.5.1 software (Camo Analytics, Oslo, Norway). OriginPro 2016 (54-bit) Sr2 b9.3.2.303 (Academic) software (OriginLab, Northampton, MA, USA) was used for graphical representation and some simple statistics.

Before applying the multivariate analysis, we applied two pre-processing procedures. First, the spectra were smoothed using a Savitzky–Golay algorithm using a fourth-order polynomial and 15 smoothing points, followed by a detrending procedure. The detrend algorithm computes a baseline function by fitting a polynomial to the sample spectrum using least squares. This process is applied to individual spectra and differs from transformations that work on each wavelength across multiple spectra. As the polynomial order increases, more baseline effects are removed, resulting in progressively refined baseline correction. We used the detrending procedure based on a second-order polynomial which corrects for offset, slope, and curvature.

In the case of PCA, we employed the leave-one-out cross-validation (LOOCV) technique to validate the model. This method involves systematically designating one sample as the validation set, utilizing the remaining samples for training. This process iterates for each sample in the dataset, guaranteeing that every data point acts as a validation set at least once. The model’s performance across all iterations is then evaluated, offering comprehensive validation by comparing each sample against all others. LOOCV proves especially beneficial for optimizing data utilization and obtaining a robust assessment of the model’s ability to generalize.

A similar approach was used for the validation of the model in the case of SVM. The cross-validation method used was an 88-segment cross-validation, which is equivalent to an LOOCV. Sequentially, one sample was tested using the classifier trained on the remaining 87 subsets, thus preventing overfitting.

## 5. Conclusions

The findings in this study underscore the potential use of ATR-FTIR on human urine, combined with multivariate and machine learning techniques, as an effective tool for distinguishing renal cell carcinoma (RCC) from healthy donors.

The spectroscopic analysis of human urine and artificial urine spiked with main urine compounds identified the key vibrational bands of urine, which are clearly dominated by urea. The spectroscopic data indicate that creatinine levels are statistically significantly lower in RCC, however, creatinine alone cannot be used as a biomarker for RCC as it is not specific, its urine concentration being altered in various kidney diseases.

Both LDA-PCA and SVM algorithms, when carefully performed to avoid overfitting, achieved good accuracy of discrimination between RCC and healthy control samples, accuracy of around 80%, with LDA-PCA showing superior discrimination figures.

ATR-FTIR spectroscopy of urine combined with machine learning shows potential for RCC screening. Still, further clinical studies and comparisons with other cancers and organ-specific diseases are required for a comprehensive understanding before this technique can be clinically applied. Additional research is necessary to accurately identify and assign the urine FTIR peaks and improve the effectiveness of this technique in cancer detection.

## Figures and Tables

**Table 1:** The main vibration bands of urine with their corresponding wavenumbers, the assignments from the literature, and those proposed in this study.

Wavenumber Measured [cm^−1^]	Wavenumber Reported [cm^−1^]	Assignments in the Literature	Tentative Assignments in this Study
513	517	CO deformation vibration (Calcium oxalate) [20]	Glucose
581	571–593	deformation vibration of the PO_4_ group (Magnesium ammonium phosphate) [20] OH libration vibrations (Calcium oxalate) [20] Strong C-H deformation [32]	Phosphate
781	756–783	CO deformation (Calcium oxalate) [20] deformation vibration of the PO_4_ group (Magnesium ammonium phosphate) [20] C–O–C stretching in Esters (Cholesterol) [20] ω N-H, C-H (ring) δ (Urea, uric acid) [33]	Urea, Uric acid
930	929–947	OH libration vibrations (Calcium oxalate) [20], ν S-O, ν P-OH (Phosphate, sulfate, nucleic acids) [33]	Phosphate
990	978–1015	ν PO_4_ (Magnesium ammonium phosphate) [20], ν_as_ (C-C), β-sheet of proteins δ(=CH) of lipids [34], ν(C-O), ν(C-C), δ(OCH) [34]	Uric acid
1075	1060–1084	P-O, NH_2_, S=O stretching (Urea, sulfate, phosphate, nucleic acids) [33] P-O, NH_2_, S=O stretching [34] CH_2_OH groups, C–O stretching and COH groups, symmetric Glycosylated proteins, PO_2_—stretching (Glycosylated proteins) [35,36,37] ν_s_ CO–O–C (Carbohydrates) [38] C–O–C stretching in Esters (Cholesterol) [20] Very Weak, Sugar ring vibration [32]	Phosphate
1118	1113–1115	C-H, C-N-C stretching (Creatinine) [33] ν (C-O), ν(C-C), δ(OCH) [34]	Creatinine
1151	1143–1157	C-NH_2_, C-O, S-O stretching (Urea, uric acid, citrate, sulfate) [33] NH_2_ deformation (Urea) [26,35], ν_s_ PO_2_^−^ [38]	Urea
1238	1230–1238	ν C-N, CH_2_ rocking (Creatinine, uric acid, citrate) [33] asymmetrical PO^−^ (nucleic acids) [38]	Creatinine
1347	1316–1399	ω CH_3_CH_2_ [39], ν C-N (Uric acid, creatinine) [33] ν_s_ COO^−^ (proteins) [20,38] ν_s_ CO (Calcium oxalate) [20], Amide III (Cholesterol) [20] Lactate, carboxylic acids and derivatives [32]	Creatinine, Uric acid
1449	1447–1468	δ CH_2_ (lipids) [20,34,38], ν_as_ (C-N) [34] δ C-H (Urea, uric acid, creatinine) [33] ν_as_ C–N (Urea) [26,35,36] CH_3_CH_2_ deformation mode [39] CH_2_ vibrations of lipids [23] ammonium ion (Magnesium ammonium phosphate) [20] δ CH_2_, δ CH_3_ ν(N=O) symmetrical deformation [32]	Urea
1604	1604–1620	N-H deformation, C-N-H vibrations (Urea) [33] ν_as_ (C-N) bending [34] ν_as_ CO group (Cystine) [20] ν_as_ (Calcium oxalate) [20] OH deformation vibration of water of crystallization (Magnesium ammonium phosphate) [20]	Urea
1652	1641–1685	Amide I [20,32,34,35,39] Random coil [35], α helix [35] 3–10 helix, π helix, type III turn [35] C=O stretching, C=N stretching (Urea, uric acid, creatinine, proteins) [33] β sheet & β turn [35]	Urea, Creatinine, Uric acid
3204	3196–3240	O–H stretching [36,37,40], Symmetric N–H stretching of Amide A [36,37,40], C–H stretch (Cholesterol) [20]	Urea, Creatinine
3338	3280–3346	ν_as_ H–O–H [35,36,40], ν N-H (Urea, creatinine) [33]	Urea
3429	3406–3461	NH group and/or OH (Cystine) [20] H_2_O or N-H [32] N-H stretching (Urea) [33]	Urea

ν—stretching vibrations, δ—bending vibrations, ω—wagging vibrations, s—symmetric, as—asymmetric.

**Table 2:** Student’s *t*-test for ATR-FTIR peak intensity between the RCC cases and healthy controls (CTRL).

Wavenumber (cm^−1^)	3429	3338	3204	1652	1604	1449	1347	1238	1151	1075	1047	930	838	771	581	515
*p*	0.112	0.176	0.086	**0.034**	0.121	0.289	**0.008**	**0.039**	0.782	0.463	0.306	0.485	0.053	0.058	0.098	0.189

*p* < 0.05 are highlighted in bold characters.

**Table 3:** Student’s *t*-test for the scores of principal components obtained in the PCA between the RCC cases and healthy controls (CTRL).

No. PC	1	2	3	4	5	6	7	8	9	10	11	12	13	14	15
*p*	0.174	0.178	0.350	**<0.001**	0.560	0.159	**0.026**	0.75	0.199	0.516	0.103	0.281	0.941	0.822	0.693

*p* < 0.05 are highlighted in bold characters and shaded areas.

**Table 4:** Accuracy of discrimination between RCC and CTRL samples using SVM and different kernel functions.

Kernel	Linear	Polynomial	RBF	Sigmoid
**Training** **accuracy (%)**	88.6	88.6	90.9	73.9
**Validation** **accuracy (%)**	76.1	76.1	71.6	63

## Data Availability

The data presented in this study are available upon reasonable request addressed to the corresponding authors. The processed data are contained within the article.

## Supplementary Materials

The following supporting information can be downloaded at: https://www.mdpi.com/article/10.3390/ijms25189830/s1.
