# Supplementary material for: Renal Cell Carcinoma Discrimination through Attenuated Total Reflection Fourier Transform Infrared Spectroscopy of Dried Human Urine and Machine Learning Techniques
Source: Int J Mol Sci. 2024 Sep 11;25(18):9830. doi: 10.3390/ijms25189830 (PMC11432727; doi:10.3390/ijms25189830)
Supplement: Supplementary file 1 [file ijms-25-09830-s001.zip › ijms-3183188-supplementay.pdf]

Supplementary Information

Renal Cell Carcinoma Discrimination through Attenuated Total Reflection Fourier Transform Infrared Spectroscopy of Dried Human Urine and Machine Learning Techniques

Bogdan Adrian Buhas [1,2,†], Lucia Ana-Maria Muntean [3,†], Guillaume Ploussard [4], Bogdan Ovidiu Feciche [2],
Iulia Andras [5], Valentin Toma [6], Teodor Andrei Maghiar [2], Nicolae Crișan [1,5], Rareș-Ionuț Știufiuc [6,7,8,*]
and Constantin Mihai Lucaciu [8,*]

[1] Department of Urology, Medicover Hospital, 323T Principala St., 407062 Suceagu, Romania;
buhasbogdan@yahoo.co.uk (B.A.B.); drnicolaecrisan@gmail.com (N.C.)
[2] Faculty of Medicine and Pharmacy, University of Oradea, 1 Universitatii St., 410087 Oradea, Romania;
dr.feciche@yahoo.com (B.O.F.); teodormaghiar@yahoo.com (T.A.M.)
[3] Department of Medical Education, Iuliu Hatieganu University of Medicine and Pharmacy,
8 Victor Babes St., 400347 Cluj-Napoca, Romania; ana.muntean@umfcluj.ro
[4] Department of Urology, La Croix du Sud Hospital, 52 Chemin de Ribaute St., 31130 Quint-Fonsegrives, France; dr.gploussard@gmail.com
[5] Faculty of Medicine, Iuliu Hatieganu University of Medicine and Pharmacy, 8 Victor Babes St.,
400347 Cluj-Napoca, Romania; dr.iuliaandras@gmail.com (I.A.);
[6] Department of Nanobiophysics, MedFuture Research Center for Advanced Medicine, Iuliu Hatieganu
University of Medicine and Pharmacy, 4-6 Pasteur St., 400337 Cluj-Napoca, Romania; valentin.toma@umfcluj.ro
[7] Nanotechnology Laboratory, TRANSCEND Research Center, Regional Institute of Oncology, 700483 Iași, Romania
[8] Department of Pharmaceutical Physics–Biophysics, Faculty of Pharmacy, Iuliu Hatieganu University of Medicine and Pharmacy, 6 Pasteur St., 400349 Cluj-Napoca, Romania; clucaciu@umfcluj.ro; rares.stiufiuc@umfcluj.ro
* Correspondences: rares.stiufiuc@umfcluj.ro (R.-I.Ș.); clucaciu@umfcluj.ro (C.M.L.); Tel.: +40-744647854 (C.M.L.)
† These authors contributed equally to this work.

Table S1. Demographic data and tumor-related information of the renal cell carcinoma patients enrolled in the study.

Number	Age (years)	Sex	Tumor histology	TNM[1]	Stage	ISUP[2] grade	Intratumoral necrosis	Intratumoral bleeding
1	68	M	Clear cell carcinoma	T2aN0M0	2	2	No	Yes
2	76	M	Clear cell carcinoma	T3aN0M0	3	4	Yes	Yes

3	73	M	Clear cell carcinoma	T1aN0M0	1	1	No	No
4	44	M	Clear cell carcinoma	T1aN0M0	1	2	No	Yes
5	73	M	Clear cell carcinoma	T3aN0M0	3	2	No	Yes
6	62	M	Clear cell carcinoma	T1bN0M0	1	2	Yes	Yes
7	56	M	Clear cell carcinoma	T1bN0M0	1	2	No	Yes
8	64	M	Clear cell carcinoma	T1aN0M0	1	1	No	No
9	77	M	Clear cell carcinoma	T2aN0M0	2	2	No	No
10	67	M	Clear cell carcinoma	T1bN0M0	1	2	No	No
11	60	M	Clear cell carcinoma	T1aN0M0	1	2	No	Yes
12	59	M	Clear cell carcinoma	T1aN0M0	1	2	No	Yes
13	72	M	Clear cell carcinoma	T2aN0M0	2	3	Yes	Yes
14	67	M	Clear cell carcinoma	T3aN0M0	3	4	Yes	Yes
15	75	M	Clear cell carcinoma	T2aN0M0	2	2	No	Yes
16	47	M	Clear cell carcinoma	T2aN0M0	2	1	Yes	Yes
17	61	M	Clear cell carcinoma	T2aN0M0	2	2	No	Yes
18	55	M	Clear cell carcinoma	T1aN0M0	1	2	No	Yes
19	61	M	Clear cell carcinoma	T1aN0M0	1	2	No	No
20	64	M	Clear cell carcinoma	T3aN0M0	3	2	Yes	Yes
21	54	M	Clear cell carcinoma	T1aN0M0	1	2	No	Yes
22	62	M	Clear cell carcinoma	T1bN0M0	1	4	Yes	Yes
23	53	M	Clear cell carcinoma	T2aN0M0	2	2	No	Yes
24	60	M	Clear cell carcinoma	T2aN0M0	2	3	No	Yes
25	64	M	Clear cell carcinoma	T1bN0M0	1	1	Yes	Yes
26	60	M	Clear cell carcinoma	T2aN0M0	2	1	No	Yes
27	44	M	Clear cell carcinoma	T1bN0M0	1	3	No	No

28	66	M	Clear cell carcinoma	T1aN0M0	1	1	No	Yes
29	38	M	Clear cell carcinoma	T1aN0M0	1	1	No	No
30	59	M	Clear cell carcinoma	T3aN0M0	3	2	Yes	Yes
31	69	M	Clear cell carcinoma	T1bN0M0	1	2	No	Yes
32	74	M	Clear cell carcinoma	T1bN0M0	1	2	Yes	Yes
33	76	M	Clear cell carcinoma	T2aN0M0	2	2	Yes	Yes
34	72	M	Clear cell carcinoma	T1aN0M0	1	2	No	Yes
35	70	M	Clear cell carcinoma	T1bN0M0	1	1	No	Yes
36	69	M	Clear cell carcinoma	T1bN0M0	1	1	Yes	Yes
37	57	M	Clear cell carcinoma	T1aN0M0	1	2	No	No
38	56	M	Clear cell carcinoma	T1bN0M0	1	1	No	Yes
39	63	M	Clear cell carcinoma	T2aN0M0	2	2	No	Yes
40	78	M	Clear cell carcinoma	T1aN0M0	1	2	No	Yes
41	64	M	Clear cell carcinoma	T3aN0M0	3	4	Yes	Yes
42	65	M	Clear cell carcinoma	T1aN0M0	1	2	No	Yes
43	56	M	Clear cell carcinoma	T3aN0M0	3	2	Yes	Yes
44	70	M	Clear cell carcinoma	T1aN0M0	1	2	No	Yes
45	50	M	Clear cell carcinoma	T1aN0M0	1	2	No	No
46	69	M	Clear cell carcinoma	T1aN0M0	1	2	No	Yes
47	41	M	Clear cell carcinoma	T1aN0M0	1	1	No	No
48	54	M	Clear cell carcinoma	T1bN0M0	1	4	Yes	Yes
49	48	M	Clear cell carcinoma	T1aN0M0	1	2	No	Yes

1-Tumor Node Metastases
2-International Society of Urologic Pathologists

Table S2. Demographic data of controls

Number	Age (Years)	Sex
1	40	M
2	57	M
3	64	M
4	79	M
5	62	M
6	48	M
7	38	M
8	84	M
9	53	M
10	73	M
11	59	M
12	66	M
13	19	M
14	31	M
15	72	M
16	74	M
17	19	M
18	83	M
19	45	M
20	73	M
21	54	M
22	65	M
23	82	M
24	68	M
25	50	M
26	60	M
27	74	M
28	73	M
29	88	M
30	62	M
31	52	M
32	70	M
33	66	M
34	62	M
35	26	M
36	64	M
37	59	M
38	51	M
39	53	M

Table S3. Age statistics for the RCC patients and the controls enrolled in this study

	N total	Mean	Standard Deviation	Minimum	Median	Maximum
Controls	39	59.43	17.1	19	62	88
RCC Patients	49	62.16	9.8	38	63.5	78

Table S4. Composition of artificial urine

	Chemical formula	Molarity (mM)	Quantity (g/100 ml)
Sodium sulfate	Na_2SO_4	11.965	0.1700
Uric acid	$C_5H_4N_4O_3$	1.487	0.0250
Trisodium citrate	$Na_3C_6H_5O_7.2H_2O$	2.450	0.0720
Creatinine	$C_4H_7N_3O$	7.791	0.0881
Urea	CH_4N_2O	249.750	1.5000
Sodium chloride	NaCl	30.053	0.1756
Ammonium chloride	NH_4Cl	23.667	0.1266
Magnesium sulfate heptahydrate	$MgSO_4.7H_2O$	4.389	0.1082
Sodium Phosphate Monobasic dihydrate	$NaH_2PO_4.2H_2O$	18.667	0.2912
Sodium Phosphate Dibasic dihydrate	$Na_2HPO_4.2H_2O$	4.667	0.0831

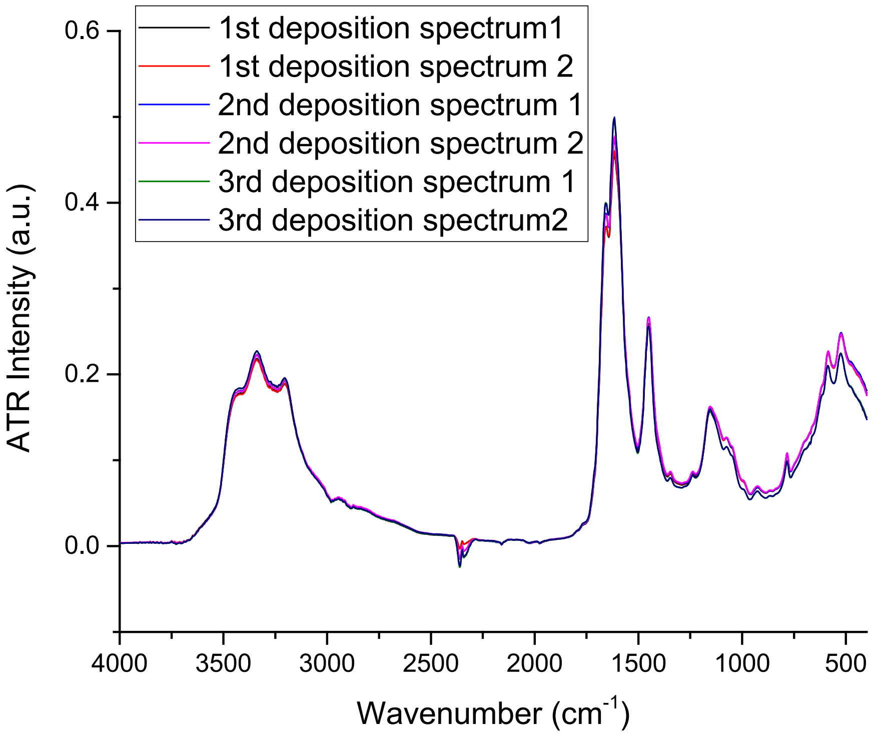

Figure S1. Example of ATR-FTIR spectra of a urine control sample were measured 6 times. For each sample 2µL of urine was deposited on the diamond crystal of the spectrometer, allowed to dry and two consecutive spectra of 16 scans each were recorded. After that, the ATR diamond was cleaned thoroughly with isopropyl alcohol, allowed to dry and another volume of 2µL of urine was deposited again. For each sample, at least three times 2mL of urine were allowed to dry and the FTIR spectra were recorded twice.

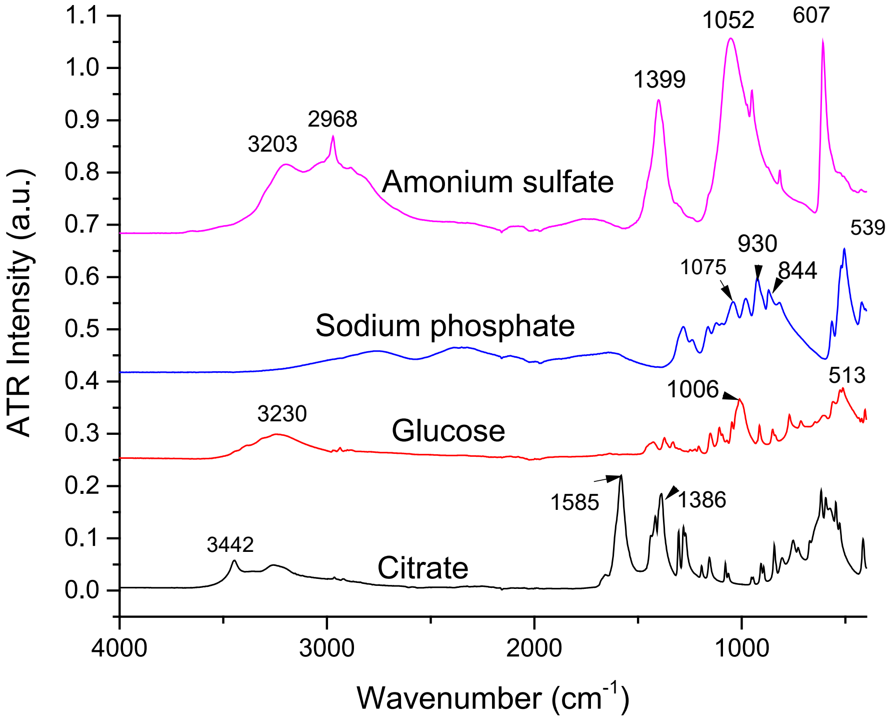

Figure S2. ATR-FTIR spectra of some urine compounds and their main absorption peaks: sodium citrate dihydrate (black), glucose (red), sodium phosphate monobasic (blue) , and ammonium sulfate (magenta).

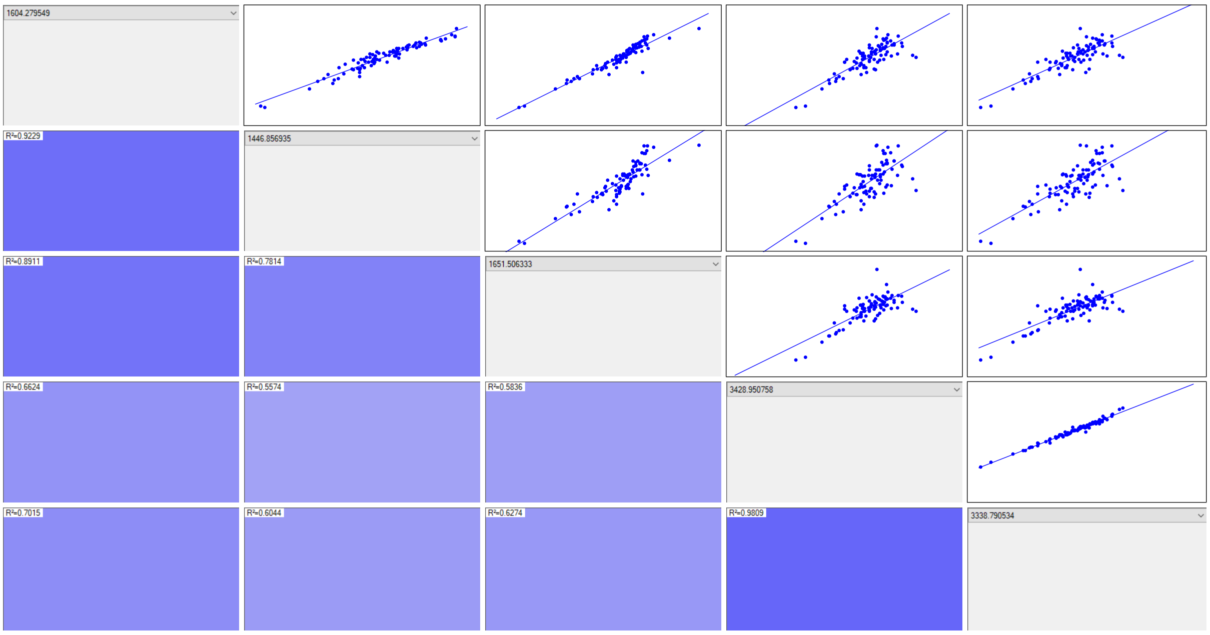

Figure S3. Correlations between the ATR-FTIR intensities measured at 1604 cm^{-1}, 1652 cm^{-1}, 1447 cm^{-1}, 3429 cm$^{-1,}$ and 3338 cm^{-1} assigned to urea.

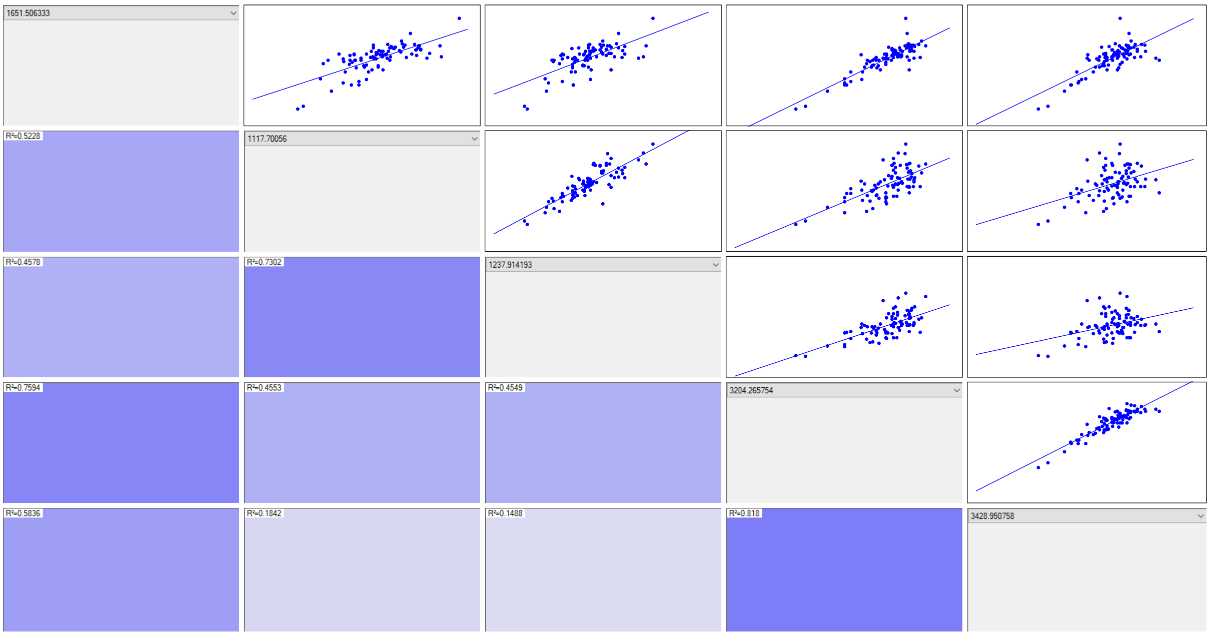

Figure S4. Correlations between the ATR-FTIR intensities measured at 1652 cm^{-1}, 1118 cm^{-1}, 1238 cm$^{-1,}$ 3204 cm$^{-1,}$ and 3429 cm^{-1}.

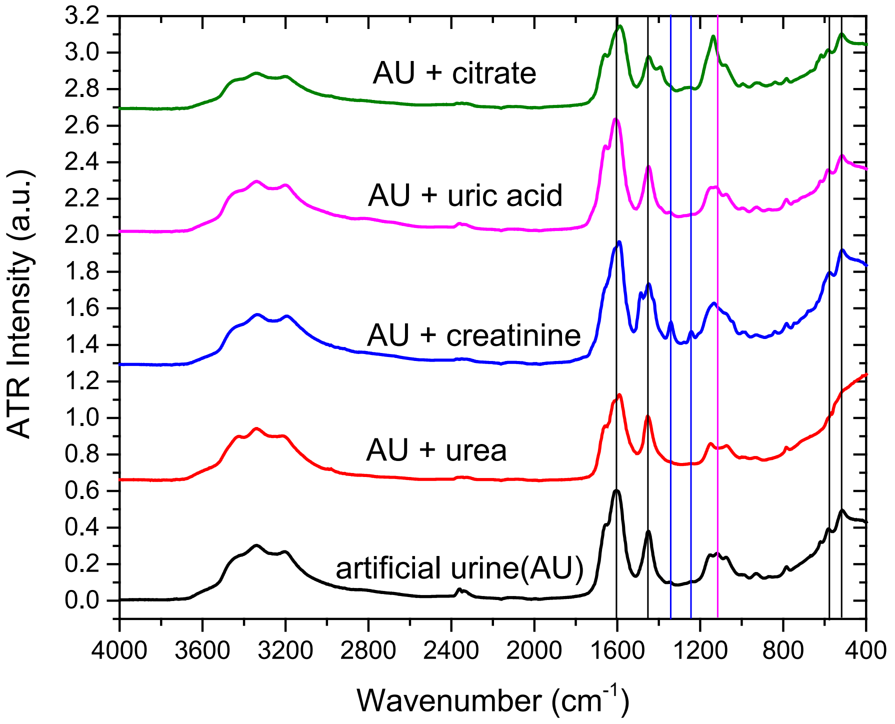

Figure S5. ATR-FTIR spectra of artificial urine (AU) and of artificial urine spiked with 10 times higher concentrations of urea (red), creatinine (blue), uric acid (magenta), and sodium citrate (green). The black vertical lines are guides for the eye to see how the maxima at 1604 cm^{-1} and 1447 cm^{-1} are shifted or not with the spiked molecules. The blue vertical lines mark the two peaks of creatinine at 1238 cm^{-1} and 1347 cm^{-1}, respectively. The black lines at 582 cm^{-1} and 581 cm^{-1}, not assigned show that they cannot be evidenced in the spectrum of urea-spiked artificial urine.

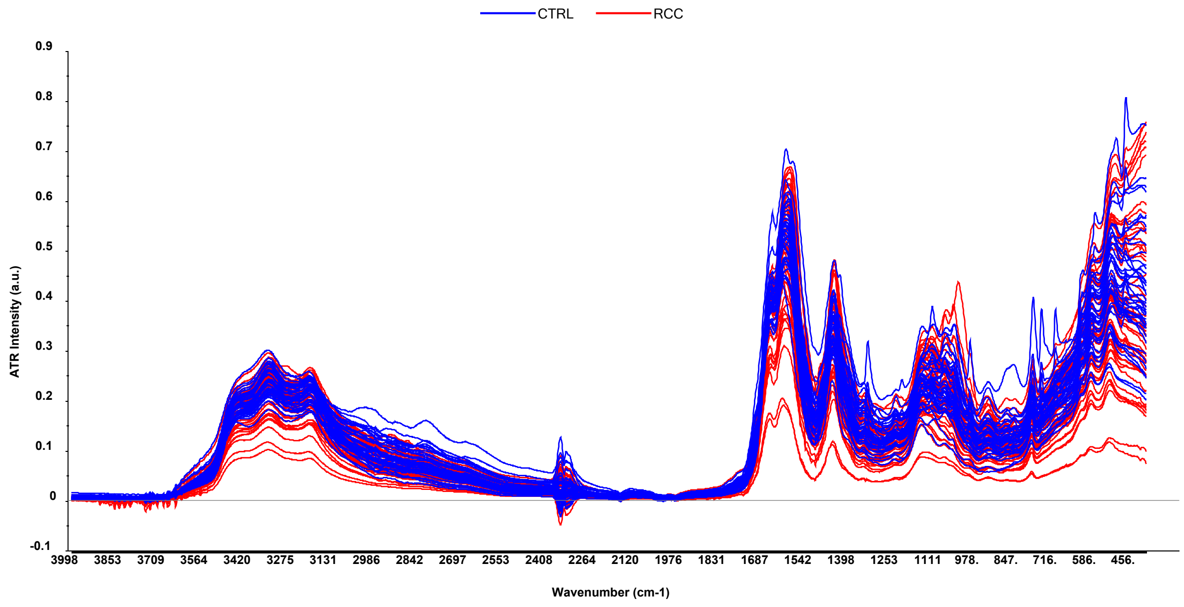

Figure S6. ATR-FTIR spectra of all samples grouped according to their categories: control (CTRL) blue and RCC red.

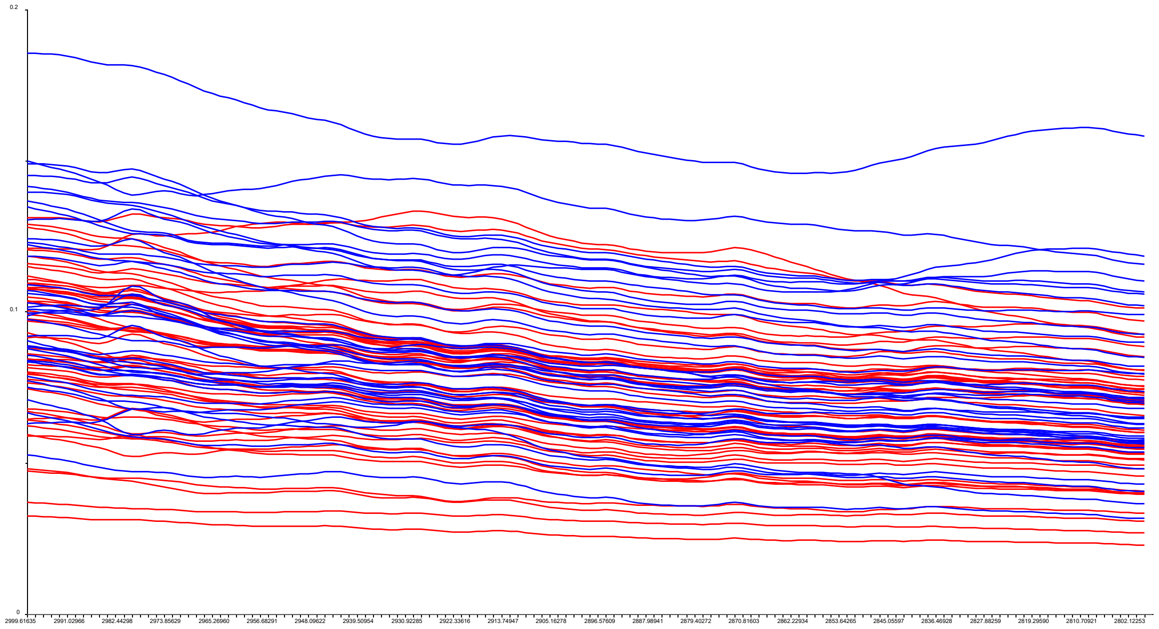

Figure S7. ATR-FTIR spectra of all samples grouped according to their categories: control (CTRL) blue and RCC red in the spectral range 2800-2900 cm^{-1}.

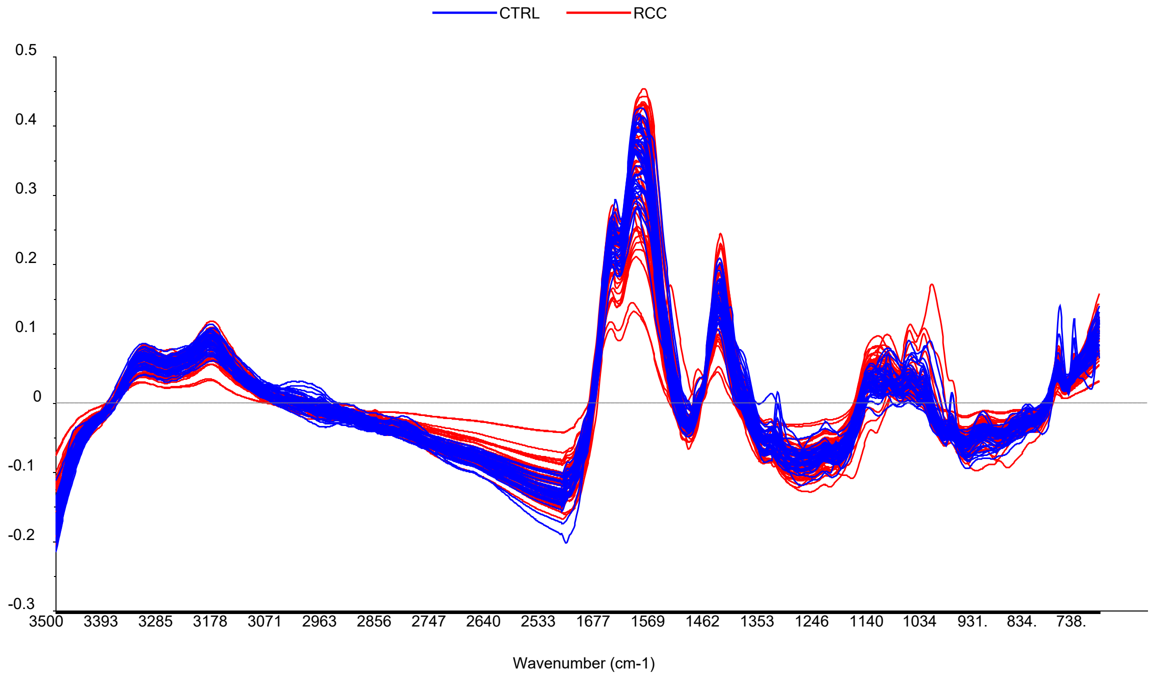

Figure S8. ATR-FTIR spectra of all samples grouped according to their categories: control (CTRL) blue and RCC red after preprocessing, i.e. smoothing with Savitzki Golay algorithm and de-trending.

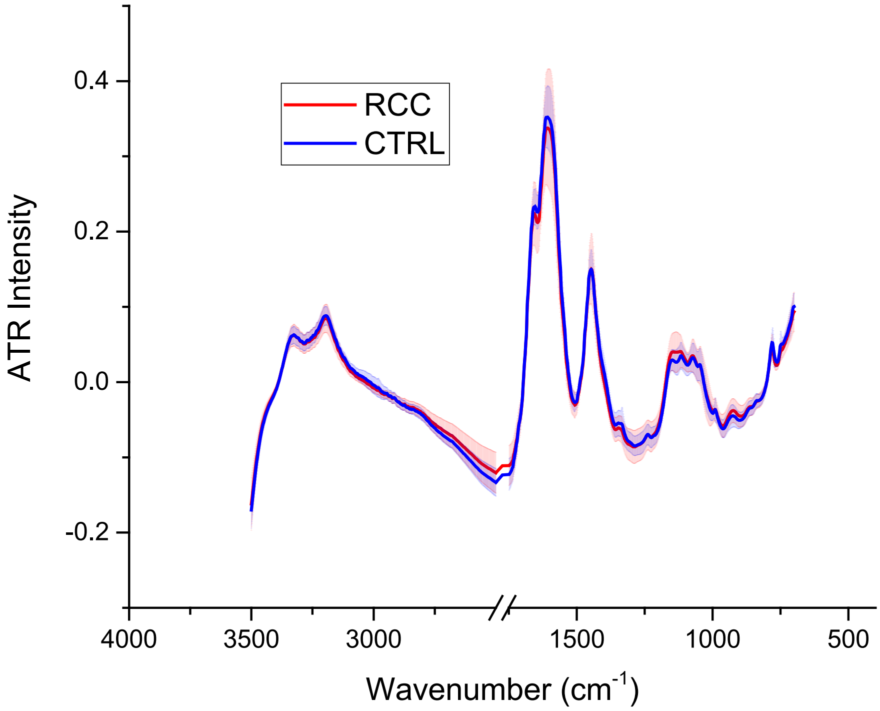

Figure S9. Mean spectra of urine samples for RCC (red) and CTRL (blue) cases after detrending. The dashed areas represent the standard deviations.

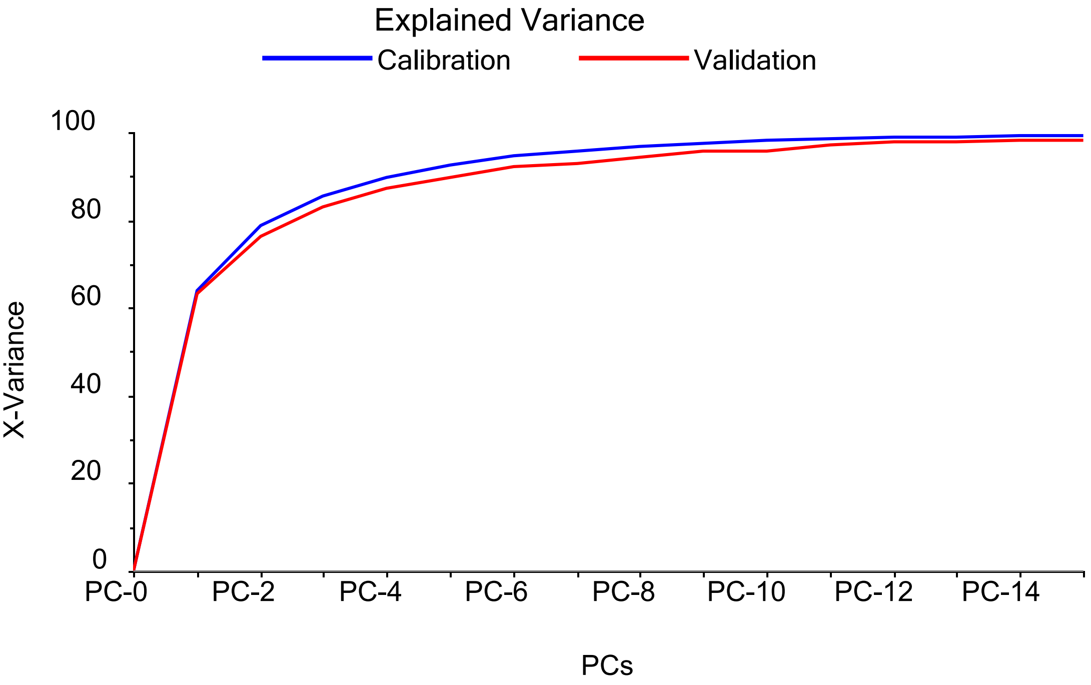

Figure S10. The cumulative explained variance for the 15 PCs, within the frame of PCA analysis on smoothed detrended data.

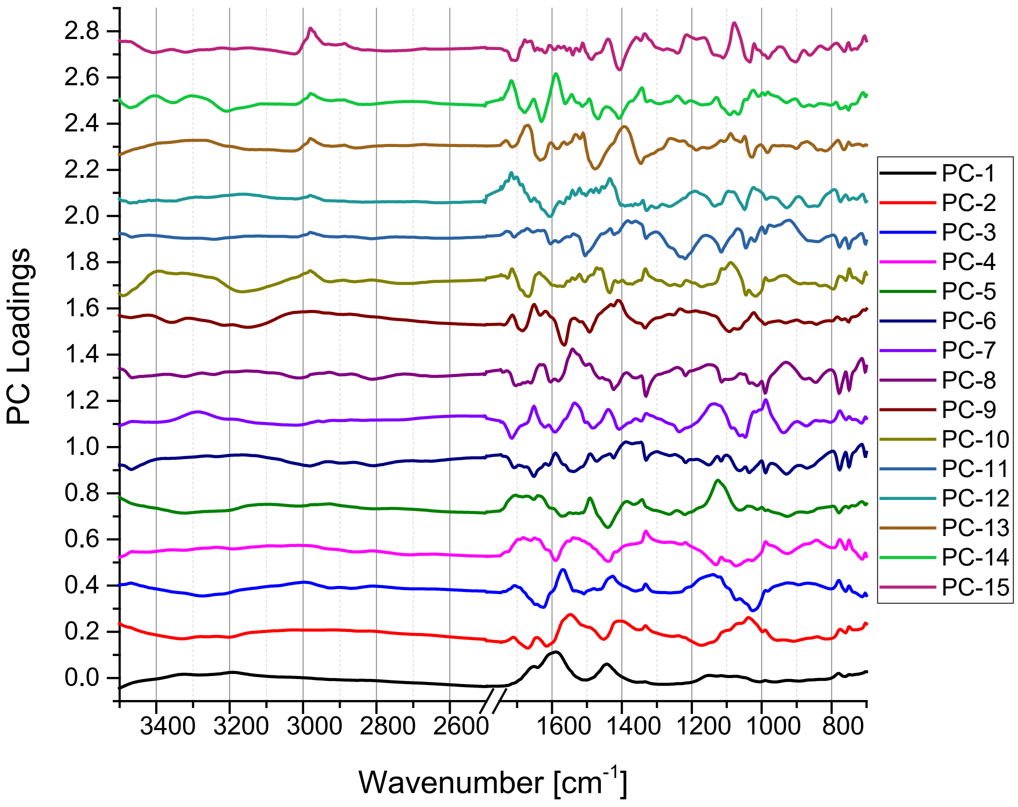

Figure S11. Loading plots of the first 15 Principal Components in the PCA.

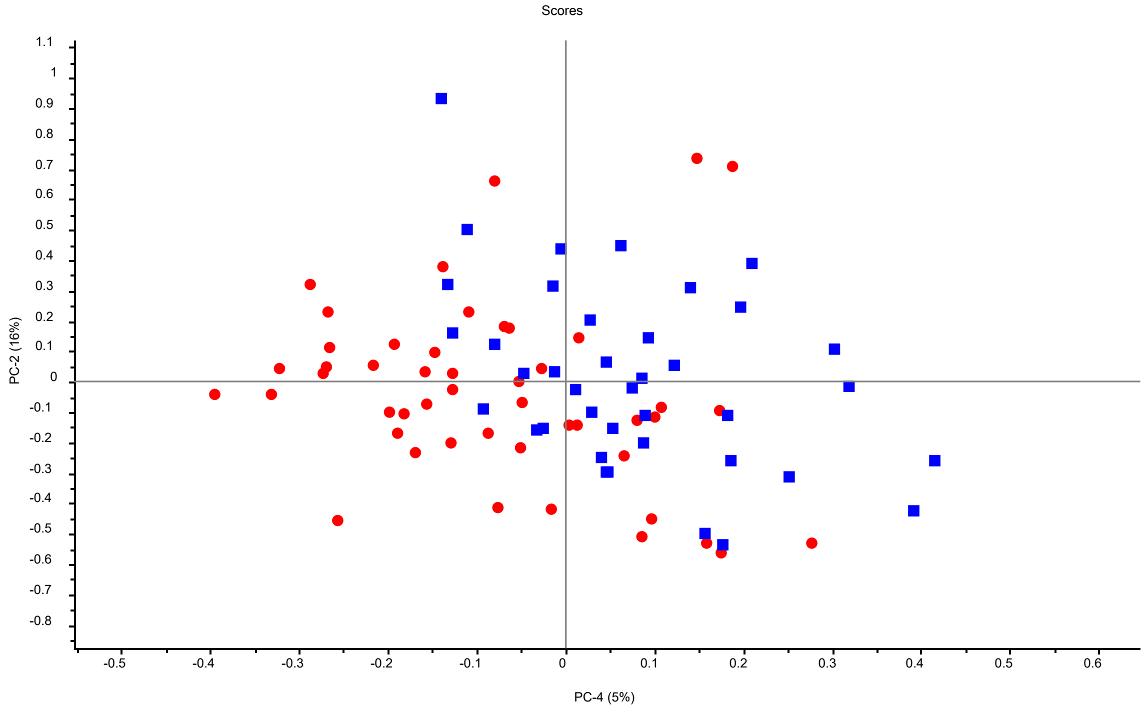

Figure S12. Scores of the RCC (red) and CTRL (blue) samples for PC4, and PC 2 showing a slight difference between the two groups.

Table S5. Confusion matrix for the discrimination between RCC and CTRL samples using QDA-PCA and considering 15 PCs.

Actual/Predicted	RCC	CTRL	Total predicted
RCC	46	4	50
CTRL	3	35	38
Total Actual	49	39	

FP-false positive; FN-false negative; TN-true negative; TP-true positive
Accuracy= TP+TN/(TP+FN+FP+FN) =92.05 %
Sensitivity= TP/(TP+FN)= 93.88 %
Specificity= TN/(TN+FP)= 89.74%

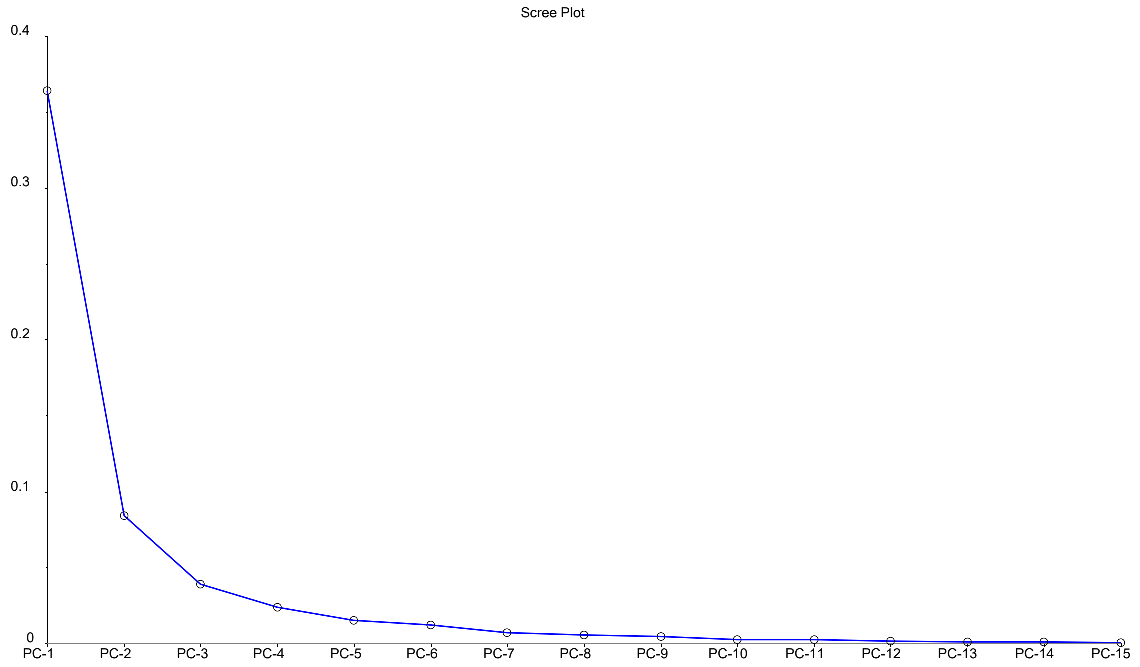

Figure S13. Scree Plot, i.e. the eigenvalues as a function of the number of PC in the PCA.
